# A Proton Pump Inhibitor Independently Elevates Gastrin Levels as a Marker for Metachronous Gastric Cancer After Endoscopic Submucosal Dissection

**DOI:** 10.3390/jcm13216599

**Published:** 2024-11-03

**Authors:** Hajime Teshima, Hidehiko Takigawa, Takahiro Kotachi, Akiyoshi Tsuboi, Hidenori Tanaka, Ken Yamashita, Yoshihiro Kishida, Yuji Urabe, Toshio Kuwai, Akira Ishikawa, Shiro Oka

**Affiliations:** 1 Department of Gastroenterology, Graduate School of Biomedical and Health Sciences, Hiroshima University Hospital, Hiroshima 734-8551, Japan; teshima@hiroshima-u.ac.jp (H.T.); kotachi@hiroshima-u.ac.jp (T.K.); atsuboi@hiroshima-u.ac.jp (A.T.); hitanaka@hiroshima-u.ac.jp (H.T.); kenyama5@hiroshima-u.ac.jp (K.Y.); kishida1@hiroshima-u.ac.jp (Y.K.); beyan13@hiroshima-u.ac.jp (Y.U.); oka4683@hiroshima-u.ac.jp (S.O.); 2 Gastrointestinal Endoscopy and Medicine, Hiroshima University Hospital, Hiroshima 734-8551, Japan; kuwai@hiroshima-u.ac.jp; 3 Department of Molecular Pathology, Graduate School of Biomedical and Health Sciences, Hiorshima University Hospital, Hiroshima 734-8551, Japan; a-ishikawa@hiroshima-u.ac.jp

**Keywords:** gastric cancer, metachronous multiple carcinomas, serum gastrin, endoscopic submucosal dissection

## Abstract

**Background and Objective:** Serum markers such as gastrin and pepsinogen are useful for stratifying gastric cancer risk. However, their utility in predicting metachronous gastric cancer after endoscopic submucosal dissection (ESD) in patients with gastric cancer after *Helicobacter pylori* eradication (GCAE) is unclear. This study aimed to clarify predictive factors for metachronous gastric cancer after ESD with a focus on serum markers. **Methods:** A retrospective analysis was conducted on 197 patients with 224 GCAE lesions who underwent ESD at Hiroshima University Hospital between April 2010 and December 2019. In total, 63 patients with 74 differentiated-type lesions were classified into metachronous gastric cancer (MG) and non-metachronous gastric cancer (NMG) groups, excluding proton pump inhibitor (PPI) users, female patients, and undifferentiated-type cases. The predictive value of serum markers was assessed using ROC curve analysis, and their association with carcinogenesis was evaluated using multiple logistic regression. Furthermore, the incidence of MG was compared between long-term PPI users and non-users. **Results:** ROC analysis revealed that serum gastrin had the highest discriminative ability for MG (AUC 0.77, cut-off 99 pg/mL, sensitivity 61.6%, and specificity 80.0%). Severe mucosal atrophy and high gastrin levels were significantly more common in the MG group and were independent predictors (*p* < 0.01). Although serum gastrin levels were significantly elevated in PPI users, no increased risk of MG was observed. **Conclusions:** In addition to severe mucosal atrophy, PPI-independent elevated serum gastrin levels may be associated with an increased risk of MG after ESD. Serum gastrin may serve as a valuable marker for post-ESD cancer surveillance in GCAE patients.

## 1. Introduction

Endoscopic submucosal dissection (ESD) for early gastric cancer has recently become a common procedure worldwide. It preserves the stomach and maintains the patient’s quality of life [1,2,3,4]. However, this approach is associated with an increased risk of gastric cancer recurrence, particularly in cases of metachronous multiple carcinomas. Previous studies have reported an annual incidence of 2.6–3.5% for metachronous multiple gastric cancers after ESD for EGC [5,6]. *Helicobacter pylori* (Hp) eradication may reduce the risk of gastric cancer development. However, gastric carcinogenesis can still occur following Hp eradication [7,8,9]. Aberrant methylation in DNA promoter regions has been reported to be involved in the molecular mechanism of gastric carcinogenesis [10]. In addition, it is believed that improvements in methylation abnormalities following eradication may inhibit carcinogenesis [11]. Recent studies have aimed to stratify the risk of gastric cancer by measuring the degree of methylation in the gastric mucosa and clarify the correlation between the degree of methylation and the risk of gastric carcinogenesis [12]. Serum gastrin and pepsinogen (PG) levels reflect the function and condition of the gastric mucosa and are considered representative biomarkers of gastric physiology [13]. Although stratification of the risk of gastric cancer using serological data has not yet been introduced in the current clinical setting, the PG1 value, PG2 value, PG1/2 ratio, and other factors are used for national screening in Japan to detect chronic gastritis [14,15,16].

The serum levels of G-17 in patients with GC were significantly higher than those in non-GC patients, indicating that individuals with higher serum G-17 levels have a higher risk of developing GC [17]. Gastrin has also been shown to stimulate the growth of human gastric cancer cell lines in cell culture [18,19]. However, some studies have not found a direct association between gastrin and gastric carcinogenesis [20], and the relationship between gastrin levels and carcinogenesis remains controversial.

Moreover, factors that can cause fluctuations in gastrin levels include proton pump inhibitor (PPI) administration, chronic kidney disease, and autoimmune gastritis [21,22]. Proton pump inhibitors (PPIs) are well-known to increase serum gastrin levels, but in some cases, gastrin levels may not serve as a predictive factor for gastritis [23,24].

Furthermore, various methods, including endoscopic findings and the histopathological examination of endoscopic biopsy specimens, are used for surveillance [25]. However, annual EGDs are expensive and physically demanding, making them a challenging routine surveillance method. Therefore, this study aimed to evaluate the usefulness of serum markers as predictors for the development of metachronous gastric cancer (MG) during surveillance after ESD for gastric cancer after eradication (GCAE).

## 2. Materials and Methods

### 2.1. Study Design

This single-center, retrospective, observational cohort study was conducted at the Hiroshima University Hospital. The study adheres to the principles of the Declaration of Helsinki. Written informed consent for the procedure was obtained from all patients. The study protocol was approved by the Ethics Committee of Hiroshima University Hospital (approval number: E2022-0295). The ethics committee waived the requirement for informed consent from each patient due to the use of anonymized data; thus, we obtained informed consent using an opt-out option.

### 2.2. Patients

We retrospectively assessed 197 patients with 224 GCAE lesions and no history of gastric cancer before successful eradication who underwent ESD at the Hiroshima University Hospital between April 2010 and December 2019. Patients with undifferentiated-type GCAE (18 patients, 18 lesions), female patients with GCAE (40 patients, 42 lesions), and autoimmune gastritis (10 patients, 11 lesions) were excluded from this study. In total, 136 male patients with differentiated-type gastric cancer and 160 lesions were selected.

**Figure 1:**
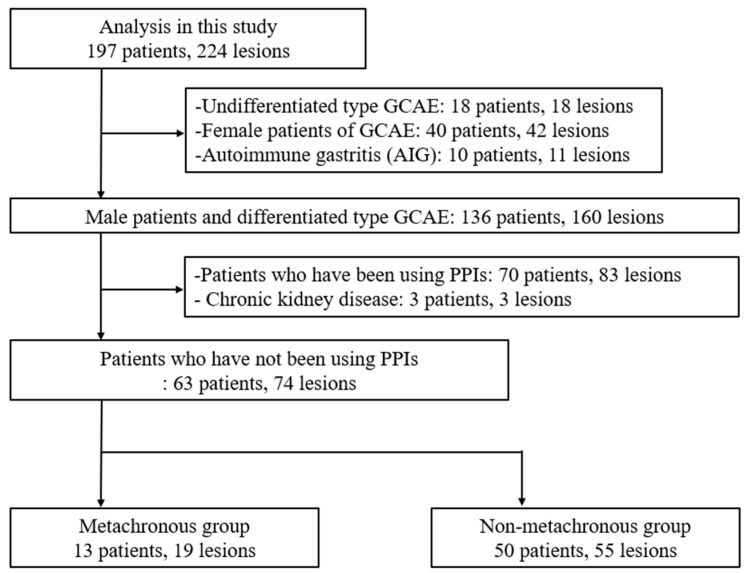
Flowchart showing the enrollment process for patients in the study, comparing the characteristics of patients with and without metachronous gastric cancer.

Subsequently, to evaluate the effect of PPI-induced fluctuations in serum markers on carcinogenesis, patients who had been using PPIs (70 patients, 83 lesions) and those with CKD (three patients and three lesions) were excluded. Finally, 63 EGC cases with 74 lesions were included in this study (Figure 1). Metachronous cancer was defined as a new tumor arising at a distant site at least 12 months after ESD.

**Figure 2:**
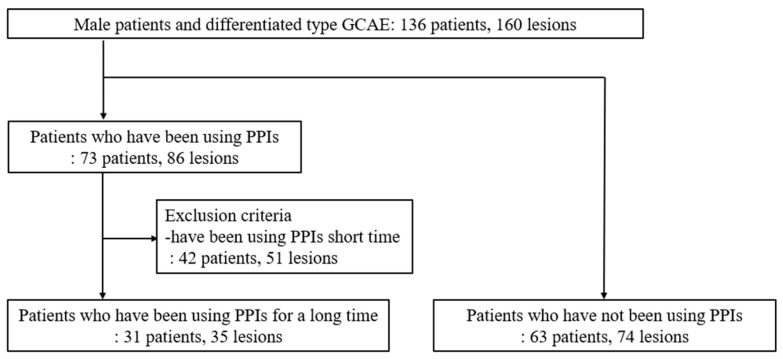
Flowchart of enrolled patients used to evaluate the influence of PPI-induced gastrin elevation on the development of metachronous gastric cancers.

Male patients with differentiated-type GCAE who did not use PPIs or have chronic kidney disease were selected for analysis. GCAE: gastric cancer after eradication; PPI: proton pump inhibitor. Subsequently, to evaluate the relationship between PPI-induced gastrin elevation and the development of MG after ESD, we compared the frequency of metachronous gastric carcinogenesis between patients who used PPIs for more than one year (31 patients, 35 lesions) and those who did not use PPIs (63 patients, 74 lesions) (Figure 2). Gastrin levels were measured at the time of ESD, and we included patients who had already started PPI therapy prior to the ESD procedure.

This flowchart shows the classification of male patients with differentiated-type GCAE lesions. Patients were categorized into groups based on long-term PPI use or no PPI use, with exclusion criteria for short-term PPI use.

### 2.3. Data Collection and Definitions

The following clinicopathological characteristics were evaluated: patient demographics (age, sex, smoking history, and family history of gastric cancer), endoscopic findings (degree of atrophy, macroscopic type, location, and color tone), and histopathological characteristics (tumor size, depth of invasion, and lymphovascular invasion).

As only male patients with differentiated gastric cancer had multiple MG after resection, we selected these patients to investigate the relationship between metachronous occurrence and serum markers (gastrin, pepsinogen, and Hp antibody titers). Gastric tumor location and gross type were classified according to the Japanese Classification of Gastric Cancer (JCGC) [26].

In this study, type 0-I (protruding) and type 0-IIa (superficial elevated) were described as “elevated”, while type 0-IIc (superficial depressed) and type 0-IIa+IIc (elevated with central depression) were described as “depressed”.

Endoscopic evaluation of atrophic gastritis was performed according to the Kimura and Takemoto classification criteria [27]. The pathological diagnosis of each tumor was made according to the JCGC criteria [26]. Smoking was defined as the regular consumption of at least five cigarettes per day.

### 2.4. Evaluation Criteria for Hp Eradication

Past eradication history was confirmed through patient interviews or medical records, and the negativity of Hp infection was confirmed using a stool antigen test (Meridian Inc., Cincinnati, OH, USA) or ^13^C-urea breath test (Otsuka Pharmaceutica, Tokyo, Japan). Patients with a history of eradication therapy and negative Hp infection were classified as having undergone Hp eradication.

### 2.5. Evaluation of Serum Markers

Fasting blood samples were collected, and serum samples were stored at −20 °C until further use for both groups. Serum gastrin concentrations were measured using the Gastrin RIA Kit II (Dynabot, Tokyo, Japan). Serum PG I and II concentrations were determined using the latex agglutination test (L-Z test; Eiken, Tokyo, Japan). Serum anti-*Hp* antibody titers were evaluated by ELISA (E-plate; Eiken Chemical, Tokyo, Japan).

### 2.6. Statistics Analysis

Data are expressed as the mean ± standard deviation for normally distributed data, while non-normally distributed data are presented as the median (interquartile range). Fisher’s exact test was used to compare qualitative variables, and the Wilcoxon rank-sum test was used to compare quantitative variables. Each serum marker risk of MG was evaluated using receiver operating characteristic (ROC) curve analysis. Due to the small sample size and limited number of outcome events, we used the stepwise method for the multivariable analysis. In addition, associations were evaluated using multiple logistic regression analyses with stepwise selection. Odds ratios (ORs) and 95% confidence intervals (95% Cls) were calculated, and *p*-values < 0.05 were considered statistically significant. All data were analyzed using JMP statistical software. 16.2.0 (SAS Institute, Cary, NC, USA).

## 3. Results

### 3.1. Clinical Characteristics of the Patients and Lesions

As presented in Table 1, 63 patients with 74 lesions (61 males; mean age 69 years) were included in the analysis. The MG group included 13 patients with 19 lesions, while the non-metachronous gastric cancer (NMG) group included 50 patients with 55 lesions. In total, 20.6% of the patients developed metachronous cancer after ESD for the first gastric cancer lesion at a median follow-up of 4.0 years. The clinical characteristics of the patients are summarized in Table 1. The mean age of the 63 patients with 74 lesions was 66 years, and all patients were male. The gross lesion type was depressed in 63 (85.2%) patients. The background gastric mucosa was severely atrophied in 40 patients (54.5%), representing half of all cases. The lesion depth was intramucosal in 68 (91.9%) patients (Table 1).

### 3.2. Comparison of Serum Markers Between MG and NMG Groups

The MG group had significantly higher serum gastrin levels (median, 109 pg/mL) compared to the NMG group (median: 75.5 pg/mL) (*p* = 0.026). The PG I/II ratio was lower in the MG group (median, 4.2) than in the NMG group (median, 5.3) (*p* = 0.036). No significant differences were observed in serum PG I or PG II levels between the two groups (Table 2).

This table compares serum gastrin and pepsinogen levels and PG I/II ratios between metachronous and non-metachronous GCAE lesions in male patients with differentiated-type lesions not using proton pump inhibitors. Significant differences were found in serum gastrin (*p* = 0.026) and the PG I/II ratio (*p* = 0.036).

### 3.3. ROC Curve Analysis for Determination of Optimal Cut-Off Values to Discriminate MG and NMG Groups

**Figure 3:**
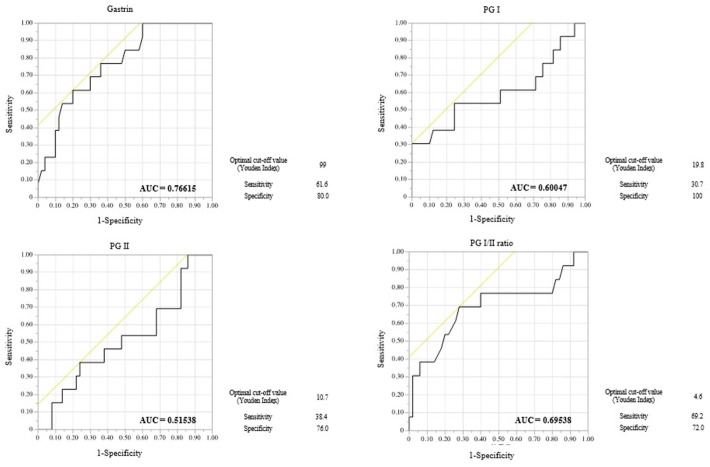
ROC curves for predictive performance of serum marker in metachronous GCAE.The yellow line in the figure serves as a reference line, representing the baseline for random classification. Positioned at a 45-degree angle, this line indicates the performance of a model with no predictive power. ROC curves that appear above this line suggest that the model’s predictive ability is better than random chance.

We conducted a comparative analysis between the MG and NMG groups and generated the corresponding ROC curves (Figure 3). The area under the curve (AUC) was largest for gastrin levels (0.76615), followed by the PG I/II ratio (0.69538), PGI (0.60047), and PGII levels (0.51538), suggesting that serum gastrin has the highest diagnostic value among these serum markers for detecting GC (Figure 3).

The optimal cut-off values for gastrin, I/II ratio, PGI, and PGII were 99 ng/mL, 4.6 ng/mL, 19.8 ng/mL, and 10.7 ng/mL, respectively. These corresponded to detection sensitivities and specificities of 61.6% and 80.0% for PGI levels, 69.2% and 72.0% for PGI levels, 30.7% and 100% for PGI levels, 38.4% and 76.0% for PG II levels, demonstrating the clinical value of gastrin for predicting MG.

This figure presents receiver operating characteristic (ROC) curves for four serum markers. Gastrin, Pepsinogen I (PG I), Pepsinogen II (PG II), and the PG I/II ratio were used to predict metachronous gastric cancer after the eradication (GCAE) of lesions. Gastrin exhibited the highest diagnostic accuracy, with an area under the curve (AUC) of 0.7661. The optimal cut-off value, determined by the Youden index, was 59, resulting in a sensitivity of 61.6% and a specificity of 80.0%.

### 3.4. Comparison of Clinical Characteristics of GCAE Between MG and NMG Groups for Predictive Factors of Metachronous Gastric Cancer

The median follow-up periods for MG and NMG were 4.0 and 4.2 years, respectively. There was no difference in the observation time between the two groups (*p* = 0.979).

Table 3 presents a comparison of the clinicopathological characteristics between MG and NMG cases. No significant differences were observed between the groups with regard to age, smoking history, family history of gastric cancer, or the number of years since eradication.

Gastric mucosal atrophy was more severe among patients in the MG group than those in the NMG group (93 vs. 46%, *p* = 0.012). However, no significant differences were observed in histology, gross type, depth, or synchronous tumors between the two groups.

Patients in the MG group exhibited higher gastrin levels than those in the NMG group (*p* < 0.01). Notably, patients in the MG group had lower PGI levels than those in the NMG group (*p* < 0.01). However, patients in the MG group had lower PG I/II ratio levels than those in the NMG group (*p* < 0.01).

Multiple logistic regression revealed that severe gastric mucosal atrophy (odds ratio [OR], 8.53; 95% confidence interval [CI], 1.47–49.4; *p* = 0.016) and high gastrin levels (OR, 8.31; 95% CI, 1.89–36.4; *p* = < 0.01) were significantly independent risk factors for the development of MG (Table 3). In a post hoc power analysis, the detection power for elevated gastrin and severe atrophy as risk factors for MG was 0.993 and 0.999, respectively; this suggests sufficient power despite the small sample size. For PG I, which has been reported in previous studies as a potential risk factor for carcinogenesis, the post hoc power was 0.658, which is relatively low.

### 3.5. Comparison of Clinical Characteristics Between PPI Group and Non-PPI Group

Patients in the long-term PPI user group had higher gastrin levels than those in the non-PPI user group (*p* < 0.01). The PPI user group tended to be older than the non-PPI user group (*p* < 0.01). Patients in the PPI group had larger tumors than those of PPI non-users (*p* = 0.036). Notably, no significant differences were observed in the incidence of metachronous cancer between the PPI users compared to patients in the PPI non-user groups (Table 4).

### 3.6. Comparison of Gastrin Value Between MG and NMG Cases Among PPI Group

We conducted a comparative analysis between the MG (3 cases) and NMG groups (28 cases) among PPI users and found no significant differences in gastrin levels between the two groups (Supplementary Table S1). ROC curve analysis showed a low AUC of 0.54762, with an optimal cut-off value of 293.0 ng/mL for gastrin in PPI users. This cut-off value corresponded to a sensitivity of 66.7% and a specificity of 71.4%. Although a higher cut-off value for gastrin was required in PPI users compared to non-PPI users, the predictive ability remained low, indicating that PPI-induced elevations in gastrin levels do not contribute to cancer prediction (Supplementary Figure S1).

## 4. Discussion

In this study, we investigated the predictive factors for the development of MG following ESD for early gastric cancer in male patients with differentiated gastric cancer. Our findings suggest that severe atrophy of the background gastric mucosa and high gastrin levels may serve as predictive markers for the development of metachronous GCAE. In addition, we found that high gastrin levels resulting from the long-term oral administration of PPIs are not associated with the development of metachronous GCAE. The serum gastrin level is included as one of the indices in the GastroPanel for predicting the development of gastric cancer [28]. Shiotani et al. identified a gastrin level of 60 pg/mL or higher as a risk factor for gastric carcinogenesis [29]. Nagasaki et al. reported that serum gastrin is a valuable marker for diagnosing gastritis according to the Updated Sydney System (USS) [30]. Another study indicated that serum gastrin levels may serve as a predictive marker for gastric cancer when comparing gastric cancer cases with non-gastric cancer cases [31].

In contrast, serum pepsinogen, a marker of atrophic gastritis included in both ABC screening and the GastroPanel [32], was not identified as an independent predictor of gastric cancer development in this study. Shiotani et al. reported that a PG1 level of 45 or less was a predictor of gastric carcinogenesis when comparing gastric cancer cases with non-gastric cancer cases [29]. However, in contrast to previous studies, the post hoc power for PG I, a potential risk factor for carcinogenesis, in our study was 0.658, which is relatively low. In this study, we compared the presence and absence of MG after endoscopic submucosal dissection (ESD) in patients with gastric cancer. We attribute the discrepancy between our findings and those of previous studies on PG1 to differences in the study populations, specifically, initial gastric cancers versus MG following ESD. These results suggest that the serum gastrin level may be a better predictor than the pepsinogen level, particularly in cases of metachronous GCAE.

Severe gastric mucosal atrophy has been consistently identified as a significant risk factor for gastric cancer, and severe atrophy is clearly associated with an increased risk of malignancy [33,34,35]. Shichijo et al. reported a strong association between severe atrophic gastritis and an elevated risk of gastric cancer [36]. Similarly, Uemura et al. identified the degree of atrophy as a crucial predictor of gastric cancer development, particularly in the context of Hp infection [37]. In this study, metachronous GCAE was associated with severe atrophy of the background gastric mucosa, which is consistent with the findings of previous studies. In addition, being male and having a history of gastric cancer have been widely recognized as significant risk factors for gastric cancer, with general consensus [38]. Women were excluded from this study due to the differences in normal gastrin levels between sexes [39]. Similarly, patients with a history of gastric cancer were excluded because this factor has a strong influence; as a result, neither sex nor a history of gastric cancer was assessed as a risk factor.

Serum gastrin levels are also elevated in patients with severe gastric mucosal atrophy [40]. In this study, severe atrophy and high gastrin levels were identified as independent risk factors through multivariate analysis. These findings underscore the importance of measuring gastrin levels as a possible predictor of metachronous GCAE risk. As previously discussed, serum gastrin is considered a marker of both gastritis and gastric cancer, and it is well-known that the oral administration of PPIs can increase gastrin levels [41,42,43]. One previous report suggested that oral administration of PPIs may contribute to carcinogenesis [44]. In recent years, meta-analyses incorporating 12 non-randomized trials and two randomized clinical trials showed negative results [45]. Regarding the PPI-induced increase in serum gastrin, one previous study reported a slight increase in the HR for carcinogenesis [46], while another did not find any associated risk, leading to ongoing controversy [41]. Physiological hypergastrinemia was identified as a risk factor for carcinogenesis, whereas PPI-induced hypergastrinemia was not. Although we initially considered severe atrophy as a potential determinant, our findings indicate that both hypergastrinemia and mucosal atrophy are independent predictors, suggesting the involvement of other factors. Previous studies have reported that gastrin levels correlate more closely with intestinal metaplasia than with atrophic gastritis [47], and intestinal metaplasia is a known risk factor for gastric cancer [48]. Therefore, physiological hypergastrinemia may reflect the presence of intestinal metaplasia, making it a predictor of cancer risk. However, since our case series did not include data on intestinal metaplasia, we were unable to verify this hypothesis.

In this study, we confirmed that atrophy and gastrin are significant factors in carcinogenesis; however, new predictive markers have also been proposed in recent years. Specifically, molecular markers such as microRNAs (e.g., miR-106) are gaining attention as important biomarkers for the mechanisms of gastric cancer development and prognosis prediction [49]. Future studies that integrate these emerging predictive markers with those used in our study may provide further insights.

We identified serum gastrin as a useful marker at the time of ESD, but post-ESD levels were not measured, limiting recommendations on timings for follow-up. Elevated gastrin, especially with severe atrophic gastritis, suggests a higher risk of metachronous cancer, but EGD remains essential. Shortening surveillance intervals may be beneficial for high-risk patients, though further evidence is required. Future studies on post-ESD gastrin levels and cancer prognosis are necessary to refine follow-up strategies.

The strength of this study lies in its focus on high-risk patients who underwent their first gastric ESD after Hp eradication. This design enabled a more precise identification of risk factors for metachronous carcinogenesis by excluding atypical cases and concentrating on well-differentiated gastric cancer, which is commonly observed after eradication.

This study has certain limitations. First, it was a small-scale analysis from a single institution, and the statistical significance of the results may change with a larger sample size. Second, the number of patients with MG was small. Although this study had the largest sample size among previous studies, further multicenter studies are needed to accumulate and examine more cases. Third, the analysis in this study was restricted to male patients, and thus, the findings are applicable only to male patients with gastric cancer. Fourth, as this study focused on patients who underwent ESD for gastric cancer to evaluate the risk of MG, a non-cancer control group was not included. Another limitation is that the assay used for measuring the total gastrin level in this study is not the only existing modality for measuring antibody titers.

## 5. Conclusions

Severe atrophy of the background gastric mucosa and high serum gastrin levels may be associated with the development of metachronous GCAE. Serum markers may be useful in predicting metachronous GCAE. However, high gastrin levels resulting from long-term PPI use did not contribute to the development of metachronous GCAE.

## Figures and Tables

**Table 1:** Baseline characteristics of gastric cancer after eradication (63 patients, 74 lesions).

Baseline Characteristics	
Total patients	63
Mean age, y. o. [range]	66 (49–89)
Mean tumor size, mm. [range]	10 (2–90)
Average observation period [year]	5.2 (2.4–11.9)
Smoking	42 (66.7)
Gastric cancer family history, *n* (%)	16 (25.4)
Location	
Upper third	16 (21.6)
Middle third	35 (47.2)
Lower third	23 (31.2)
Macroscopic type	
Superficial elevated	11 (14.8)
Superficial depressed	63 (85.2)
Gastric mucosal atrophy	
Mild	4 (5.4)
Moderate	30 (40.1)
Severe	40 (54.5)
Invasion depth	
Mucosa	68 (91.9)
Submucosa	6 (8.1)
Metachronous tumor	
Negative	50 (79.4)
Positive	13 (20.6)
Synchronous tumor	
Negative	49 (77.8)
Positive	14 (22.2)
Serum gastrin (pg/mL)	78 (31–222)
Serum PG I (ng/mL)	42 (8.2–157.7)
Serum PG II (ng/mL)	8.1 (3.8–27.6)
PG I/II ratio	5.1 (0.8–9.4)

PG: pepsinogen (%).

**Table 2:** Serum gastrin and pepsinogen levels and *H. pylori* antibodies of males with differentiated-type lesions undergoing metachronous and non-metachronous GCAE who had not been using proton pump inhibitors.

Variables	Metachronous (+), 13 Patients 19 Lesions	Metachronous (−), 50 Patients 55 Lesions	*p-*Value
Serum gastrin (pg/mL)	109 (70–222)	75.5 (31–157)	0.026 *
Serum PG I (ng/mL)	28.8 (8.2–110.1)	42 (20.3–157.7)	0.757
Serum PG II (ng/mL)	8.2 (5.2–16.5)	8 (3.8–27.6)	0.709
PG I/II ratio	4.2 (0.8–7.8)	5.3 (1.7–9.4)	0.036 *

GCAE: gastric cancer after eradication; PG, pepsinogen; *: a significant difference.

**Table 3:** Comparison of the clinical characteristics of the GCAE primary lesion between the metachronous and non-metachronous groups.

Variables	Metachronous		Univariate Analysis	Multivariate Analysis	
	(+), 13 Patients, 19 Lesions	(−), 50 Patients, 55 Lesions	*p*-Value	OR (95% CI)	*p*-Value
Age (years)			0.616	−	−
<70	10 (77)	35 (70)			
≥70	3 (23)	15 (30)			
Smoking	11 (85)	20 (62)	0.104	−	−
Gastric cancer family history	7 (23)	13 (26)	0.827	−	−
Eradication period (years)			0.332	−	−
<10	11 (85)	36 (72)			
≥10	2 (15)	14 (28)			
Gastric mucosal atrophy			0.012	8.53 (1.47–49.4)	0.016 *
Mild/moderate	2 (7)	27 (54)			
Severe	11 (93)	23 (46)			
Tumor size (mm)			0.235	−	−
≤10	3 (16)	16 (29)			
>10	16 (84)	39 (71)			
Tumor location			0.261	−	−
Upper/middle	15 (79)	36 (65)			
Lower	4 (21)	19 (35)			
Macroscopic type			0.12	−	−
Superficial elevated	5 (26)	6 (11)			
Superficial depressed	14 (74)	49 (89)			
Invasion depth			0.181	−	−
Mucosal	16 (84)	52 (95)			
Submucosal	3 (3)	3 (5)			
Synchronous tumor			0.439	−	−
Negative	9 (70)	44 (81)			
Positive	4 (30)	10 (19)			
Serum gastrin (pg/mL)			<0.01 *	8.31 (1.89–36.4)	<0.01 *
<99	5 (38)	40 (80)			
≥99	8 (62)	10 (20)			
Serum PG I (ng/mL)			<0.01 *	−	−
<19.8	4 (31)	0 (0)			
≥19.8	9 (69)	50 (100)			
Serum PG II (ng/mL)			0.622	−	−
<10.7	9 (69)	38 (76)			
≥10.7	4 (31)	12 (24)			
PG I/II ratio			<0.01 *	−	−
<4.6	9 (69)	14 (28)			
≥4.6	4 (31)	36 (72)			

(%) GCAE, gastric cancer after eradication; CI, confidence interval; OR, odds ratio. *: a significant difference.

**Table 4:** Comparison of the clinical characteristics of gastric cancer after the eradication of primary lesions and serum gastrin and pepsinogen levels between the PPI and non-PPI groups.

Variables	PPI (+), 31 Patients 35 Lesions	PPI (−), 63 Patients 74 Lesions	*p* Value
Gastrin (pg/mL)			<0.01 *
<99	3 (9)	45 (72)	
≥99	28 (91)	18 (28)	
PG I/II ratio			0.359
<4.6	14 (45)	22 (36)	
≥4.6	17 (55)	41 (64)	
Age (years)			<0.01 *
<70	8 (26)	45 (71)	
≥70	23 (74)	18 (29)	
Smoking	20 (65)	42 (67)	0.836
Gastric cancer family history	8 (25)	16 (25)	0.53
Eradication period (years)			0.965
<10	23 (74)	47 (75)	
≥10	8 (26)	16 (25)	
Gastric mucosal atrophy			0.707
Mild/moderate	13 (42)	29 (46)	
Severe	18 (58)	34 (54)	
Tumor size (mm)			0.036 *
≤10	16 (46)	19 (26)	
>10	19 (54)	55 (74)	
Tumor location			0.097
Upper/middle	24 (69)	51 (85)	
Lower	11 (31)	23 (15)	
Macroscopic type			0.09
Superficial elevated	10 (29)	11 (15)	
Superficial depressed	25 (71)	63 (85)	
Invasion depth			0.641
Mucosa	33 (94)	67 (93)	
Submucosa	2 (6)	7 (7)	
Synchronous tumor			0.701
Negative	27 (87)	53 (84)	
Positive	4 (13)	10 (16)	
Metachronous			0.183
(+)	3 (10)	13 (21)	
(−)	28 (90)	50 (79)	

(%) PPI, Proton pump inhibitor. *: a significant difference.

## Data Availability

The data supporting the findings of this study are available from the corresponding author upon reasonable request.

## Supplementary Materials

The following supporting information can be downloaded at: https://www.mdpi.com/article/10.3390/jcm13216599/s1, Supplementary Figure S1. ROC Curves for Predictive Performance of Gastrin in PPIs users. Supplementary Table S1. Serum gastrin levels of male and differentiated type between metachronous and non-metachronous GCAE who have been using proton-pump inhibitors.
