# Supplementary material for: A Proton Pump Inhibitor Independently Elevates Gastrin Levels as a Marker for Metachronous Gastric Cancer After Endoscopic Submucosal Dissection
Source: J Clin Med. 2024 Nov 3;13(21):6599. doi: 10.3390/jcm13216599 (PMC11546463; doi:10.3390/jcm13216599)
Supplement: Supplementary file 1 [file jcm-13-06599-s001.zip › Supplementary Figure S1.pptx]

## Slide 1
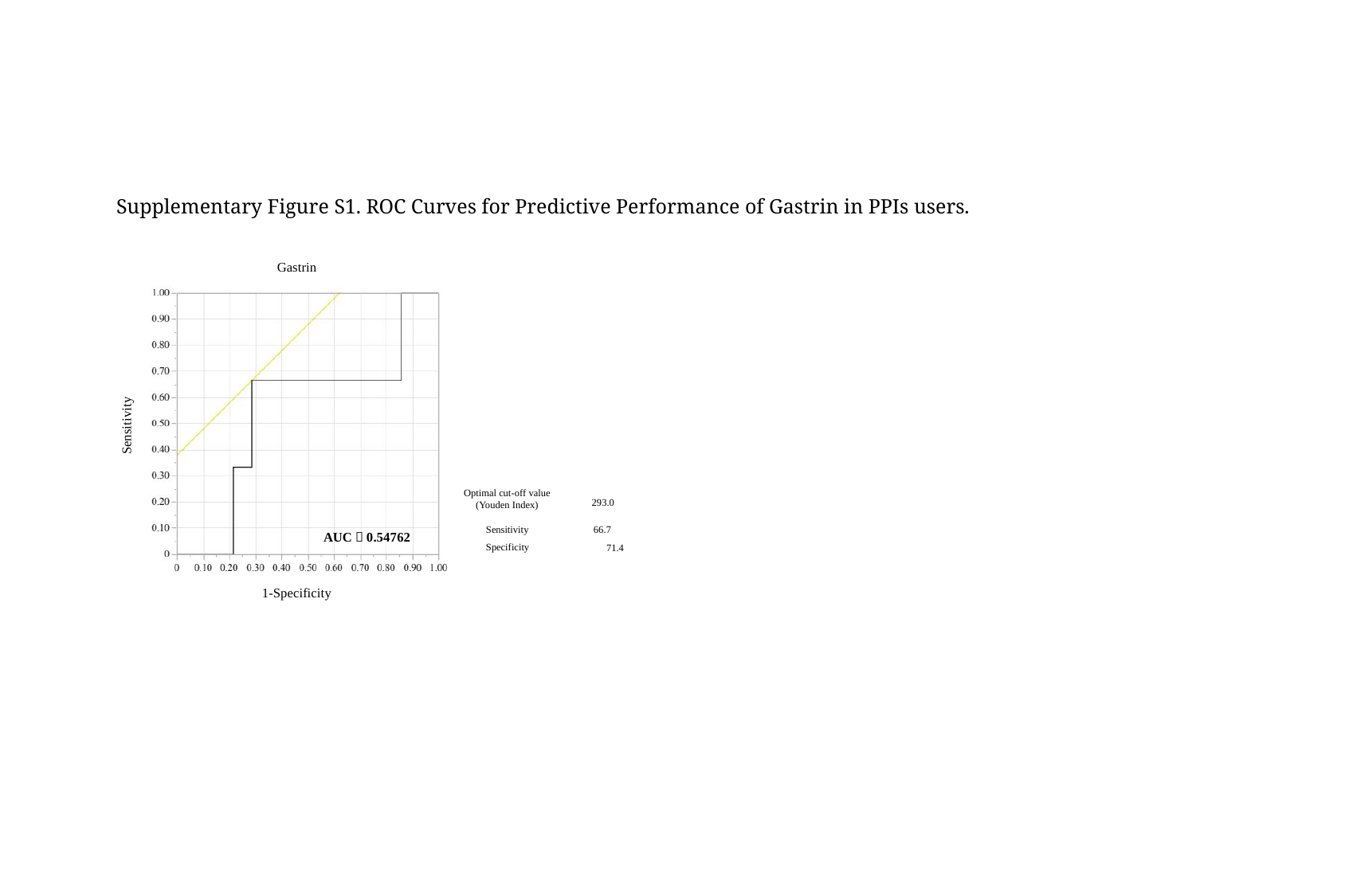

Supplementary Figure S1. ROC Curves for Predictive Performance of Gastrin in PPIs users.
Gastrin
Sensitivity
Optimal cut-off value
(Youden Index)
293.0
| | |
| --- | --- |
| Sensitivity | 66.7 |
| Specificity | 71.4 |
| | |
| | |
AUC＝0.54762
1-Specificity
