# Supplementary material for: A Proton Pump Inhibitor Independently Elevates Gastrin Levels as a Marker for Metachronous Gastric Cancer After Endoscopic Submucosal Dissection
Source: J Clin Med. 2024 Nov 3;13(21):6599. doi: 10.3390/jcm13216599 (PMC11546463; doi:10.3390/jcm13216599)
Supplement: Supplementary file 1 [file jcm-13-06599-s001.zip › Supplementary Table S1.pdf]

Supplementary Table S1. Serum gastrin levels of male and differentiated type between metachronous and non-metachronous GCAE who have been using proton-pump inhibitors.

Variables	Metachronous		*P* -value
	(+), 3 patients 3 lesions	(-), 28 patients 32 lesions	
Serum Gastrin (pg/mL)	293 (138-458)	181.5 (105-658)	0.512
